# Assessing the effects of iron enrichment across holobiont compartments reveals reduced microbial nitrogen fixation in the Red Sea coral *Pocillopora verrucosa*

**DOI:** 10.1002/ece3.3293

**Published:** 2017-07-31

**Authors:** Nils Rädecker, Claudia Pogoreutz, Maren Ziegler, Ananya Ashok, Marcelle M. Barreto, Veronica Chaidez, Carsten G. B. Grupstra, Yi Mei Ng, Gabriela Perna, Manuel Aranda, Christian R. Voolstra

**Affiliations:** ^1^ Red Sea Research Center Division of Biological and Environmental Science and Engineering (BESE) King Abdullah University of Science and Technology (KAUST) Thuwal Saudi Arabia

**Keywords:** coral reefs, diazotroph, holobiont, nutrient limitation, *Symbiodinium*, symbiosis

## Abstract

The productivity of coral reefs in oligotrophic tropical waters is sustained by an efficient uptake and recycling of nutrients. In reef‐building corals, the engineers of these ecosystems, this nutrient recycling is facilitated by a constant exchange of nutrients between the animal host and endosymbiotic photosynthetic dinoflagellates (zooxanthellae), bacteria, and other microbes. Due to the complex interactions in this so‐called coral holobiont, it has proven difficult to understand the environmental limitations of productivity in corals. Among others, the micronutrient iron has been proposed to limit primary productivity due to its essential role in photosynthesis and bacterial processes. Here, we tested the effect of iron enrichment on the physiology of the coral *Pocillopora verrucosa* from the central Red Sea during a 12‐day experiment. Contrary to previous reports, we did not see an increase in zooxanthellae population density or gross photosynthesis. Conversely, respiration rates were significantly increased, and microbial nitrogen fixation was significantly decreased. Taken together, our data suggest that iron is not a limiting factor of primary productivity in Red Sea corals. Rather, increased metabolic demands in response to iron enrichment, as evidenced by increased respiration rates, may reduce carbon (i.e., energy) availability in the coral holobiont, resulting in reduced microbial nitrogen fixation. This decrease in nitrogen supply in turn may exacerbate the limitation of other nutrients, creating a negative feedback loop. Thereby, our results highlight that the effects of iron enrichment appear to be strongly dependent on local environmental conditions and ultimately may depend on the availability of other nutrients.

## 1 INTRODUCTION

Tropical coral reefs are among the most productive and diverse ecosystems on the planet. They provide essential goods and services such as fisheries, income from tourism, and coastal protection (Moberg & Folke, 1999). Even though they occur in oligotrophic waters, coral reefs are characterized by a high primary productivity, a contradiction known as “Darwin's paradox” (Darwin, 1842; Sammarco, Risk, Schwarcz, & Heikoop, 1999). The mutualistic endosymbiotic relationship between reef‐building (hermatypic) corals and their dinoflagellate algae of the genus *Symbiodinium* (zooxanthellae) are the functional basis of this productivity. Indeed, the algal symbionts translocate most of their photosynthetically fixed carbon to the coral, enabling the animal host to thrive in highly nutrient‐poor waters (Muscatine & Porter, 1977). The coral host in turn provides the algal symbionts with nutrients from its metabolism, thereby enabling a highly efficient uptake and recycling of nutrients (Falkowski, Dubinsky, Muscatine, & Porter, 1984; Muscatine, Falkowski, Porter, & Dubinsky, 1984).

Besides the photosynthetic algal symbionts, other microbial organisms are associated with corals, such as Bacteria, Archaea, and Fungi. This assemblage has been termed the coral holobiont (Knowlton & Rohwer, 2003; Rohwer, Seguritan, Azam, & Knowlton, 2002; Rosenberg, Koren, Reshef, Efrony, & Zilber‐Rosenberg, 2007). The microbes associated with corals have essential roles in holobiont health and function, such as nitrogen cycling (Falkowski et al., 1984; Lesser, 2004; Rädecker, Pogoreutz, Voolstra, Wiedenmann, & Wild, 2015), sulfur cycling (Raina, Dinsdale, Willis, & Bourne, 2010; Raina, Tapiolas, Willis, & Bourne, 2009), and the production of antimicrobial compounds (Castillo, Lodeiros, Nunez, & Campos, 2000; Ritchie, 2006). Thus, environmental conditions that affect the composition or abundance of the associated microbiota could have significant effects on the coral host's performance and health (Ainsworth & Gates, 2016; Reshef, Koren, Loya, Zilber‐Rosenberg, & Rosenberg, 2006; Rosenberg et al., 2007). Therefore, understanding the interactions within this meta‐organism framework is vital to predict the response of corals to environmental change.

Primary productivity in corals appears to be limited by nitrogen availability (Marubini & Davies, 1996; Muller‐Parker, McCloskey, Hoegh‐Guldberg, & McAuley, 1996; Muscatine et al., 1989). Along with the uptake of inorganic nitrogen from seawater and heterotrophic feeding, dinitrogen (N_2_)‐fixing Bacteria and Archaea constitute a significant source of nitrogen within the coral holobiont (Bednarz, Grover, Maguer, Fine, & Ferrier‐Pagès, 2017; Benavides et al., 2016; Lesser et al., 2007). At the same time individual reports exist which point toward an iron limitation of coral symbionts (Entsch, Sim, & Hatcher, 1983; Ferrier‐Pagès, Schoelzke, Jaubert, Muscatine, & Hoegh‐Guldberg, 2001; Rodriguez, Lin, Ho, & Ho, 2016). Iron is a critical micronutrient in cell biology, and iron protein clusters are essential to electron transfer in most metabolic reactions, including photosynthesis and microbial N_2_ fixation (Price, 1968). Thereby, iron is a central nutrient to the cellular machinery of both processes, and iron availability controls large‐scale dynamics of both carbon and nitrogen cycling in the open ocean (Chisholm & Morel, 1991; Tagliabue et al., 2017). In the tropical surface waters surrounding coral reefs, iron is typically found at low concentrations below 1 nmol/L (Blain, Bonnet, & Guieu, 2008; Gordon, Coale, & Johnson, 1997). Given its importance as a micronutrient, it is thus critical to investigate the role of iron in coral holobiont nutrient cycling. Yet, only few studies have attempted to disentangle the role of iron, both at the organismal and the ecosystem scale.

To date, two studies reported that increasing iron concentrations can lead to higher *Symbiodinium* growth rates, both *in vitro* at up to 1 nmol/L Fe (Rodriguez et al., 2016) and *in hospite* in the pocilloporid coral *Stylophora pistillata* at 6 nmol/L Fe (Ferrier‐Pagès et al., 2001). However, the latter study found that iron enrichment was also accompanied by reduced coral skeletal growth rates, suggesting detrimental effects on the physiological performance of the coral animal.

Field observations from coral reefs have reported a negative impact of iron enrichment or investigated iron toxicity at much higher concentrations. Briefly, iron leaching from shipwrecks has been associated with the proliferation of invasive Corallimorpharia and benthic fleshy algae (Kelly et al., 2014; Schroeder, Green, DeMartini, & Kenyon, 2008; Work, Aeby, & Maragos, 2008). Additionally, excess iron concentrations (179 and 895 nmol/L Fe) were observed to decrease *Symbiodinium* densities in the scleractinian *Porites lutea*, suggesting a toxicity effect resulting in the disruption of the coral—algal symbiosis (Brown, 1989). Interestingly however, coral holobionts can evidently adapt to chronic iron exposure, as reflected in naturally higher symbiont densities and a diminished response to experimental iron enrichment (Brown, 1989). Therefore, the effect of iron enrichment on coral holobiont physiology may be largely context‐dependent.

Consequently, further studies are required to enhance our understanding of the role of iron in coral holobiont functioning. In this context, the Red Sea is a highly oligotrophic ocean system with periodically high inputs of iron via desert dust deposition (Chase, Paytan, Johnson, Street, & Chen, 2006; Jickells, 2005). To assess whether iron is limiting primary production and microbial processes in Red Sea corals, we conducted a 12‐day aquaria enrichment experiment with the common coral *Pocillopora verrucosa* from the central Red Sea. Briefly, we quantified the effects of excess iron availability on coral holobiont photosynthesis and its associated microbial nitrogen fixation activity.

## 2 MATERIALS AND METHODS

### 2.1 Coral rearing, experimental setup, and sample collection

Six adult colonies of the brown color morph of *P. verrucosa* were collected at the nearshore reef Shaab in the central Red Sea, Saudi Arabia (N22°12′02.30″, E38°59′59.55″) at a depth of 3–5 m. Colonies were sampled at least 5 m apart to ensure that different genotypes were collected (Robitzch, Banguera‐Hinestroza, Sawall, Al‐Sofyani, & Voolstra, 2015). The Saudi Coastguard Authority, under the auspices of the King Abdullah University of Science and Technology (KAUST), issued sailing permits to the site that included coral collection. The coral *P. verrucosa* is listed as “least concern” on the IUCN Red List (http://www.iucnredlist.org/details/133197/0; accessed February 2017).

**Figure 1:**
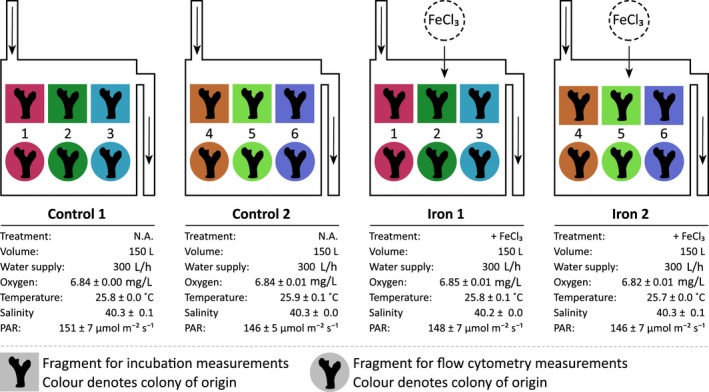
Overview of experimental setup and seawater parameters of individual tanks after 12 days of experiment. A total of six coral colonies were assessed, distributed over two control (Control 1, Control 2) and two experimental (Iron 1, Iron 2) treatment tanks. The latter received iron enrichment in the form of 900 nmol FeCl_3_ addition (6 nmol/L aquarium volume) every 30 min. Each aquarium contained fragments of three colonies (indicated by fragment background color). One fragment of each colony per treatment was used for incubation (rectangular shape) and flow cytometry (round shape) measurements, respectively. One colony was excluded from analysis of incubation measurements due to technical problems with gas chromatography measurements. All tanks were supplied with ambient reef water. High turnover rates of seawater produced stable and consistent conditions across all tanks

Colonies were transferred to the wet laboratory facility of the Coastal and Marine Resources Core Lab (CMOR) at KAUST and fragmented. A total of 24 fragments (four fragments per colony, mean individual fragment surface area = 37.2 ± 3.5 cm^2^) were each attached to 47 × 47 mm stone tiles with epoxy putty (AquaStik, Doctors Foster and Smith, USA) and acclimated for 5 days in four 150‐L flow‐through aquaria, which were each individually continuously supplied with Red Sea reef water. High seawater turnover rates (renewal rate of 300 L/h) were used to stabilize environmental parameters in all aquaria units and to avoid tank specific differences (seawater temperature at 25°C, salinity of 40, photosynthetic active radiation of ~150 μmol photons s^−1^ m^−2^ on a 12:12‐hr day/night cycle; see Figure 1 for details on experimental design and environmental parameters in aquaria tanks). After the acclimation, fragments were re‐distributed over the experimental tanks so that each treatment condition (control and iron enrichment) contained two fragments of each colony (i.e., genotype). During the experiment, two tanks served as untreated controls and two tanks were enriched with 6 nmol Fe(III)chloride per liter of aquarium volume every 30 min to achieve continuous iron enrichment. Thereby, the level of iron enrichment was in the same order of magnitude as previous studies reporting stimulated *Symbiodinium* growth and productivity under these conditions (Ferrier‐Pagès et al., 2001; Rodriguez et al., 2016). After 12 days of iron enrichment, all coral fragments from both control and treatment tanks were collected. One replicate fragment of each colony was used for incubation measurements (*n* = 6 per treatment; described in detail below), and the other was snap‐frozen in liquid nitrogen for further analyses of the *Symbiodinium* community (*n* = 6 per treatment). In this context, Pogoreutz, Rädecker, Cardenas, Voostra, & Wild (2017) showed that aquaria maintenance did not alter microbiome composition or activity in the Red Sea coral *P. verrucosa* over the course of several weeks.

### 2.2 *Symbiodinium* flow cytometry

*Symbiodinium* cells were isolated from coral tissue by NaOH extraction (Zamoum & Furla, 2012). Frozen coral fragments were thawed and incubated in 1 mol/L NaOH at room temperature for 1 hr. After the incubation period, the skeleton was removed, and suspended *Symbiodinium* cells were spun down in a bench‐top centrifuge for 3 min at 3,000 RCF. The supernatant was discarded and the *Symbiodinium* pellet was washed in 1 ml of PBS (1×). Subsequently, the pellet was resuspended in PBS with 0.01% SDS buffer. Suspended cell samples were diluted 1:10 and directly used for flow cytometry analysis (BD LSRFortessa, BD Biosciences, USA) to assess concentration of *Symbiodinium* cells, relative cell chlorophyll content, and relative cell sizes.

### 2.3 Photosynthetic efficiency

To understand the effect of iron enrichment on photosynthetic efficiency of photosystem II in *Symbiodinium*, pulse amplitude modulated (PAM) fluorometry was used. Maximum quantum efficiency (*F*_*v*_/*F*_*m*_) of each fragment was measured after 30 min dark acclimation with a Diving‐PAM fluorometer and a 6‐mm fiber optic cable (Walz, Germany). After dark acclimation, initial fluorescence (*F*_*o*_) was measured by applying a red (650 nm) measuring light. Subsequently, a saturating pulse (actinic light <710 nm, 0.8 s, >3,000 μmol m^−2^ s^−1^) was applied, and maximum fluorescence (F_m_) was measured directly afterwards. The maximum quantum efficiency was calculated as:

$$F_{v}/F_{m}\;=\;\left(F_{m}\;-\;F_{o}\right)/F_{m},$$

with *F*_*v*_ as variable fluorescence. For each fragment (*n* = 6 per treatment), three replicate measurements were conducted on different upward‐facing sides of the branches, and the mean of the replicate measurements was calculated for each fragment before statistical analysis.

### 2.4 N_2_ fixation

Following PAM fluorometry, coral fragments were used for N_2_ fixation measurements (calculated indirectly from acetylene (C_2_H_2_) reduction assays (Wilson et al., 2012)). For the assay, the six fragments (i.e., one originating from each of the six colonies) per treatment were transferred to 1‐L gas tight glass chambers for incubation. In addition, duplicate seawater control chambers were incubated to correct for planktonic N_2_ fixation activity. Each chamber contained 720 ml of seawater from the respective tank and 80 ml of C_2_H_2_‐enriched seawater. Of the 200 ml air headspace, 10% was replaced with C_2_H_2_ gas. The 24‐hour incubation was conducted in the experimental aquaria to maintain constant temperature. 2.5‐ml gas samples were collected at the beginning and at the end of the incubation from the air head space with a glass syringe and injected into blood collection tubes. Ethylene (C_2_H_4_) concentrations in the gas samples were determined using the 7890A GC system with Agilent HP‐AL/S column (Agilent Technologies, USA) and flame ionization detection. As one gas sample from the iron treatment was lost during GC analysis, the corresponding fragment as well as the counterpart colony fragment from the control treatment was therefore omitted from further analysis.

Rates of N_2_ fixation were subsequently quantified indirectly from C_2_H_4_ evolution rates without conversion. The rates were calculated based on C_2_H_4_ concentration differences between the start and endpoint of the incubation according to Breitbarth, Mills, Friedrichs, and Laroche (2004). Measured C_2_H_4_ concentrations were corrected for the respective seawater controls and normalized to incubation time and coral surface area (Rädecker, Meyer, Bednarz, Cardini, & Wild, 2014).

### 2.5 Photosynthesis and respiration

Following the C_2_H_2_ reduction assay, coral net photosynthesis and respiration were measured during 2‐hr light and dark 1‐L glass chamber incubations, respectively. Briefly, oxygen (O_2_) evolution and consumption were quantified based on differences in O_2_ concentrations before and after each incubation using an optical oxygen multiprobe (WTW, Germany). Readings were recorded after thoroughly stirring the seawater around each coral fragment. Measured O_2_ production/consumption rates were corrected for respective seawater controls and normalized to incubation time and coral surface area. Gross photosynthesis was calculated according to the equation: gross photosynthesis = net photosynthesis + respiration.

### 2.6 Surface area determination

Physiological parameters, that is *Symbiodinium* counts and rates of respiration, photosynthesis, and N_2_ fixation, were normalized to the surface area of each coral fragment. Coral surface areas were determined by creating 3D models of each individual fragment using the software Remake v.117.25 (67) (Autodesk Inc., USA). Each model was created based on approximately 30 digital photographs taken in indoor lighting.

### 2.7 Statistical analysis

To ensure that observed patterns were not influenced by tank effects, we tested the effect of individual tanks on each of the measured response parameters. For this, we used linear mixed effect models with tank identity as fixed effect and colony identity as random effect (Lindstrom & Bates, 1988). No significant tank effect was observed for any of the response parameters, and accordingly, the effect of the individual aquaria units was not included as a variable for the further analysis (Table S1). Differences between control and treatment of coral surface and time corrected measurements were tested for significance using paired Student's t tests to account for colony identity of fragments with a significance level (α) of 0.05. All data are presented as mean ± standard error.

## 3 RESULTS

### 3.1 Stable *Symbiodinium* population under iron enrichment

**Figure 2:**
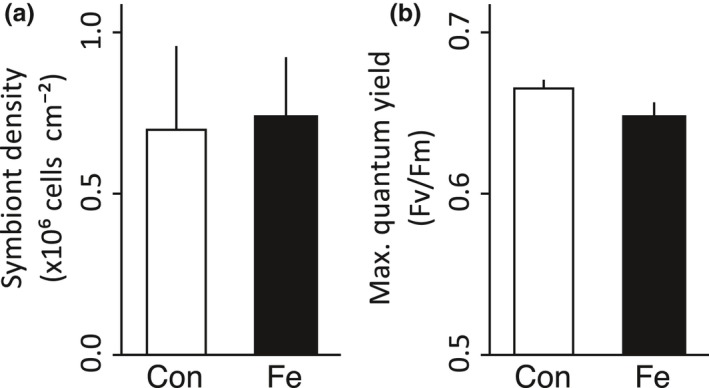
*Symbiodinium* density (a) and photosynthetic efficiency (b) in the coral *P. verrucosa* after 12 days under control (white) and iron‐enriched (black) conditions (*n* = 6 replicate colony fragments each for control and iron‐enriched conditions). Data are shown as mean ± standard error. Con, control; Fe, iron enriched

The number of *Symbiodinium* cells per coral fragment area was not significantly different (*t*_(11)_ = 0.22, *p* = .83) between the control and iron‐enriched conditions, averaging 7.01 ± 2.58 × 10^5^ and 7.39 ± 1.87 × 10^5^ cells/cm^2^, respectively (Figure 2a). These cell counts are in line with common estimates of *Symbiodinium* density in Red Sea *P. verrucosa* (Sawall, Al‐Sofyani, Banguera‐Hinestroza, & Voolstra, 2014; Ziegler, Roder, Büchel, & Voolstra, 2014). Further, relative cell size distribution and chlorophyll cell content were not statistically different between control and iron‐enriched conditions after 12 days (*t*_(11)_ = 0.50, *p* = .63 and *t*_(11)_ = 0.05, *p* = .96, respectively). However, a slightly decreased maximum photosynthetic efficiency for iron‐enriched coral colonies was observed, although this was not significantly different between the treatments (*t*_(11)_ = 1.80, *p* = 0.09, Figure 2b).

### 3.2 Iron enrichment affects respiration and net photosynthesis

**Figure 3:**
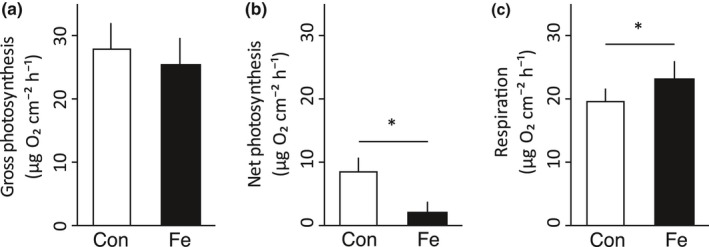
Gross photosynthesis (a), Net photosynthesis (b), and respiration (c) rates in the coral *P. verrucosa* after 12 days under control (white) and iron‐enriched (black) conditions (*n* = 5 replicate colony fragments each for control and iron‐enriched conditions). Data are shown as mean ± standard error. Significant differences between treatments are marked with asterisks (*p* < .05). Con, control; Fe, iron enriched

We observed no significant effect of iron enrichment on gross photosynthesis (28.0 ± 4.0 and 25.5 ± 4.3 μg O_2_ hr^−1^ cm^−2^ for control and iron‐enriched conditions, respectively; *t*_(9)_=1.41, *p* = .05, Figure 3). Conversely, respiration rates increased significantly by 19% (from 19.6 ± 2.1 to 23.3 ± 3.0 μg O_2_ hr^−1^ cm^−2^; *t*_(9)_ = 2.30, *p* < .05). Consequently, the largest difference was observed in net photosynthesis which decreased by 74% under iron enrichment (from 8.4 ± 2.2 to 2.2 ± 1.6 μg O_2_ hr^−1^ cm^−2^; *t*_(9)_=2.32, *p* < .05).

### 3.3 Reduced microbial N_2_ fixation under iron enrichment

**Figure 4:**
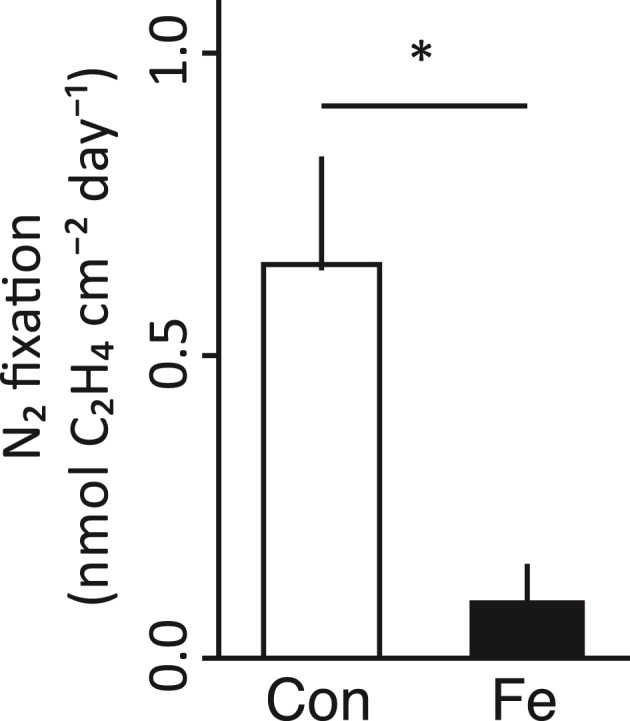
N_2_ fixation rates (expressed as ethylene (C_2_H_4_) evolution rates) in the coral *P. verrucosa* after 12 days under control (white) and iron‐enriched (black) conditions (*n* = 5 replicate colony fragments each for control and iron‐enriched conditions). Data are shown as mean ± standard error. Significant differences between treatments are marked with asterisks (*p* < .05). Con = control, Fe = iron enriched

Under iron enrichment, we found that N_2_ fixation in corals was significantly reduced by an order of magnitude after 12 days (*t*_(9)_ = 2.61, *p *<* *.05). On average, ethylene evolution rates were 0.65 nmol C_2_H_4_ day^−1^ cm^−2^ in the control and 0.09 nmol C_2_H_4_ day^−1^ cm^−2^ in the iron‐enriched treatment, representing an overall reduction in N_2_ fixation rates by 86% (Figure 4).

## 4 DISCUSSION

Despite the importance of iron as a micronutrient, surprisingly few studies have investigated its functional importance in hermatypic corals. Previous studies support the overall limiting role of iron on coral productivity, as reflected by increased photosynthesis and *Symbiodinium* cell densities under iron‐enriched conditions (Entsch et al., 1983; Ferrier‐Pagès et al., 2001; Rodriguez et al., 2016). Similarly, iron limitation may lead to an impairment of the photosynthetic apparatus of heat‐stressed *Symbiodinium* (Shick et al., 2011). In the present study, however, we found stable *Symbiodinium* densities and photosynthesis, despite increased iron levels and experimental time frames comparable to those used by Ferrier‐Pagès et al. (2001). Also, we observed increased holobiont respiration and decreased N_2_ fixation. Taken together, these findings suggest that primary production in *P. verrucosa* coral holobionts from the Red Sea are not limited by the availability of iron or by iron alone.

Furthermore, increased respiration rates indicate increased energy demands possibly leading to reduced availability of organic carbon in the coral holobiont. This in turn may lead to subsequent carbon limitation of energy‐demanding physiological processes, such as N_2_ fixation (McNarry & Burris, 1962; Rädecker et al., 2014). Thereby, the observed reduction in N_2_ fixation rates in the present study can potentially be attributed to an increased energy limitation within the coral holobiont under iron‐enriched conditions. Similarly, other studies have reported on reduced coral growth rates under iron enrichment, suggesting a potential detrimental effect on the holobiont (Brown, 1989; Ferrier‐Pagès et al., 2001).

Ultimately, the effects of increased iron availability on coral physiology must be interpreted in the context of the regional availability of other nutrients. The corals investigated in the present study were collected and reared in highly oligotrophic Red Sea waters. Dissolved inorganic nutrient concentrations for the region of the collection site are <0.4 μmol/L of inorganic nitrogen, <0.1 μmol/L of phosphate, and <0.6 μmol/L of silicate (Roik et al., 2016; Ziegler, Roder, Büchel, & Voolstra, 2015). In contrast to the low concentrations of these macronutrients, the dissolved iron concentration in the Red Sea is relatively high. In the surface waters of the Gulf of Aqaba and the Northern Red Sea, iron levels of 1.8–30 nmol/L were reported (Chase et al., 2006; Shriadah, Okbah, & El‐Deek, 2004), which is about an order of magnitude higher compared to the Western Mediterranean (≤0.13–5.0 nmol/L; (Sarthou & Jeandel, 2001) and open ocean systems (0.2–0.8 nmol/L (Johnson, Gordon, & Coale, 1997; Jickells, 2005). These differences can likely be attributed to regional atmospheric iron input from dust deposition. Indeed, dust deposition is a major iron source for ocean systems, and the regional variation of iron deposition is highly and seasonally dependent on the wind velocity and precipitation (Chase et al., 2006; Jickells, 2005; Shriadah et al., 2004). Annual iron deposition rates in the Gulf of Aqaba were reported to be four‐fold to 10‐fold higher than those in the Sargasso Sea (Jickells, 2005). Hence, corals in the Red Sea are likely not iron‐limited as opposed to corals in more iron‐depleted seawater (Ferrier‐Pagès et al., 2001). Instead, increased iron availability may exacerbate the limitation of other nutrients under these conditions, as previously reported for phosphate in corals under excess nitrogen conditions (Pogoreutz et al., 2017; Rosset, D'Angelo, & Wiedenmann, 2015; Rosset, Wiedenmann, Reed, & D'Angelo, 2017; Wiedenmann et al., 2012). Hence, the observation of increased respiration may point toward higher energetic demands of the coral holobiont to buffer and/or prevent nutrient starvation. This effect in turn may be exacerbated by the observed reduction of N_2_ fixation under these conditions, thereby creating a negative feedback loop on holobiont productivity.

Taken together, our study suggests that the effects of iron in the coral holobiont may be multifaceted and depend on the prevailing environmental conditions. Ambient iron availability (Brown, 1989) as well as the availability of other nutrients (Rodriguez et al., 2016) may determine the physiological performance of the holobiont. To disentangle the complexities of environmental nutrient availability, future work should therefore target the effects of selective removal or addition of specific nutrients as well as their combined effects. Finally, the iron seawater chemistry strongly depends on environmental conditions. Future ocean scenarios, such as ocean warming and acidification, may drastically alter iron availability in the tropical ocean (Shi, Kranz, Kim, & Morel, 2012; Shi, Xu, Hopkinson, & Morel, 2010). Consequently, further efforts are required to disentangle the role of iron in the response of corals to environmental change.

## CONFLICT OF INTEREST

None declared.

## AUTHOR CONTRIBUTIONS

NR, CP, MZ, MA, and CRV conceived and designed the experiment. All authors helped with conducting the experiment, interpreting data, and drafted and revised the article. All authors approved the final article.

## DATA ACCESSIBILITY

Data available from the Dryad Digital Repository: http://dx.doi.org/10.5061/dryad.n50jf
