# Supplementary material for: Assessing the effects of iron enrichment across holobiont compartments reveals reduced microbial nitrogen fixation in the Red Sea coral Pocillopora verrucosa
Source: Ecol Evol. 2017 Jul 31;7(16):6614–21. doi: 10.1002/ece3.3293 (PMC5574852; doi:10.1002/ece3.3293)
Supplement: Supplementary file 1 [file ECE3-7-6614-s001.docx]

**Table S1.** Summary of model statistics investigating the effect of tank identity (Control 1, Control 2, Iron 1, Iron 2) on individual response parameters. Each model consisted of a linear mixed effect model assigning tank ID as a fixed effect and colony identity as random effect. DF = degrees of freedom.

| **Response parameter** | ***DF*** | ***n*** | ***F-value*** | ***P*** |
| --- | --- | --- | --- | --- |
| Symbiont density | 3 | 12 | 0.41 | 0.763 |
| Symbiont size (rel.) | 3 | 12 | 0.18 | 0.904 |
| Symbiont chlorophyll (rel.) | 3 | 12 | 8.95 | 0.102 |
| Maximum quantum yield | 3 | 12 | 2.16 | 0.272 |
| Gross photosynthesis | 3 | 10 | 3.92 | 0.210 |
| Net photosynthesis | 3 | 10 | 12.14 | 0.077 |
| Respiration | 3 | 10 | 2.81 | 0.273 |
| N_2_ fixation | 3 | 10 | 3.48 | 0.231 |
